# Trace‐Element Incorporation into Intracellular Pools Uncovers Calcium‐Pathways in a Coccolithophore

**DOI:** 10.1002/advs.201700088

**Published:** 2017-07-05

**Authors:** Assaf Gal, Sanja Sviben, Richard Wirth, Anja Schreiber, Benedikt Lassalle‐Kaiser, Damien Faivre, André Scheffel

**Affiliations:** ^1^ Max‐Planck Institute of Molecular Plant Physiology Potsdam‐Golm 14476 Germany; ^2^ Max‐Planck Institute of Colloids and Interfaces Potsdam‐Golm 14476 Germany; ^3^ GeoForschungsZentrum Potsdam Potsdam 14473 Germany; ^4^ Synchrotron Soleil Gif‐sur‐Yvette 91192 France

**Keywords:** acidocalcisomes, biomineralization, coccolith, *Emiliania huxleyi*, X‐ray absorption spectroscopy

## Abstract

Many organisms form minerals from precursor phases that crystallize under strict biological control. The dynamic intracellular processes of formation, transport, and deposition of these precursor phases are challenging to identify. An unusual situation is recently revealed for the calcifying alga *Emiliania huxleyi*, as the cells contain a compartment filled with a concentrated Ca and P phase but the final calcite crystals, which are nucleated in a different compartment, are P‐free. Thus, the connection of the Ca–P‐rich pool to the mineralization process remains unclear. Here, pulse‐chase experiments are used with Sr to label the Ca–P‐rich phase in *E. huxleyi* cells, and cryo X‐ray absorption spectroscopy and analytical transmission electron microscopy to follow the Sr within cells. It is found that Sr is first found in the Ca–P‐rich phase and then becomes incorporated into the calcite. This demonstrates that the calcium used by the cells to build calcite originates from the Ca–P‐rich pool.

Mineral formation by organisms is often a complex, multistep process, occurring under tight biological control.^1^, ^2^ One widespread strategy is to precipitate transient amorphous precursor phases, which during the mineralization process will be sequentially modified into the final crystalline phase.^3^, ^4^, ^5^ Typically, the amorphous phase undergoes minor compositional changes. Most common is dehydration, and sometimes the stoichiometry or the oxidation state of the constituent ions changes.^6^, ^7^, ^8^ In several organisms, intracellular membrane‐bound pools containing constitutes of the mature mineral phase but also additional elements have been observed, rendering the overall composition of the pool very different from that of the mature mineral phase.^9^, ^10^, ^11^, ^12^, ^13^ Whether these pools contribute to the formation of the mature mineral phase or are unrelated entities is challenging to clarify.

One of the most remarkable examples for biomineralization is coccoliths, calcitic scales produced by a group of marine unicellular algae known as coccolithophores.^12^, ^13^, ^14^, ^15^ Each coccolith is an array of nanometer sized calcite crystals, assembled inside a specialized compartment, subsequently referred to as coccolith vesicle. Upon completion, the coccolith is extruded to the cell surface and assembled with other coccoliths into a shell enveloping the cell.^15^, ^16^

Coccolith biomineralization contributes significantly to the long term sequestration of carbon, as coccoliths sediment to the ocean floor after the algal cells die. The cellular processes underlying coccolith formation remain largely elusive. This leads to uncertainties in predicting the effect of future climate changes on coccolithophore calcification, and reconstituting past environmental conditions from trace element and isotopic signatures of coccolith calcite.^17^, ^18^, ^19^

The pathways by which the Ca ions move after their uptake inside the cells into the coccolith vesicle are virtually unknown for coccolithophores. An exception is the coccolithophore *Pleurochrysis carterae*. For this alga, coccolith calcium was shown to be transported in complexes with acidic polysaccharides into the coccolith vesicle, where macromolecular recognition directs them to the site of crystallization.^20^, ^21^ In a previous study, we identified a membrane‐bound Ca‐pool distinct from the coccolith vesicle in cells of the world's most abundant calcifier, the coccolithophore *Emiliania huxleyi*.^13^ This pool, termed Ca–P‐rich body hereafter, is also rich in phosphorus and contained other cations. Most remarkably, it lacked detectable amounts of carbonate as is contained in the widespread transient precursor phase of biocalcite, amorphous calcium carbonate (ACC). The characteristics of the Ca‐pool, which are unexpected for a precursor phase of calcite, namely, the presence of P and absence of carbonate, and its similarity to widespread organelles called acidocalcisomes that serve mineralization‐unrelated functions,^22^ raised the question if the Ca–P‐rich body is a calcium source for coccolith formation.

In this study, we aim at following the material transfer between intracellular pools in *E. huxleyi* cells, thus specifically addressing the relation between the Ca–P‐rich body and the coccolith formation. We used Sr ions, as they are considered to be transported by Ca channels and are a common impurity in coccolith calcite.^23^, ^24^ Strontium L_2,3_‐edge X‐ray absorption near edge spectroscopy (XANES) was used as a tool to follow the changes in the local environment of Sr during coccolith calcite formation. Together with transmission electron microscopy (TEM) imaging, our results show that the Sr ions are first present in the disordered phase of the Ca–P‐rich body, and are later incorporated into the coccolith calcite. Therefore, material is transferred from the Ca–P‐rich pool into the coccolith vesicle, and the Ca–P‐rich body is a component of the coccolith biogenesis pathway.

We collected Sr L_2,3_‐edge XANES spectra from synthetic reference materials in order to characterize the spectral features of Sr in the environments relevant for the coccolithophore cell (**Figure** **1**A). When Sr ions are in a solution (as in 1 × 10^−3^ m SrCl_2_), incorporated into amorphous calcium carbonate (Sr‐doped ACC), or into amorphous calcium phosphate (Sr‐doped ACP), the L_3_ and L_2_ electronic transitions are characterized by single sharp peaks. However, when Sr is incorporated into the lattice of synthetically grown calcite the XANES spectrum changes. The most pronounced difference is a split in both the L_3_ and the L_2_ peaks. This splitting corresponds to transitions from the 3p to the 4d valence levels, which are better defined when Sr is in a strict octahedral coordination sphere, such as in crystalline calcite, as compared to the distorded‐octahedra structure found in amorphous or solution samples. A spectrum similar to that of synthetic calcite was acquired from snap frozen cells, prepared by the same methodology described below, which had formed their entire coccolith shell in a medium where the [Sr] was elevated tenfolds relative to seawater ([Sr] = 600 × 10^−6^ m, [Ca] = 10 × 10^−3^ m) and thus incorporated large amounts of Sr into the calcite of coccoliths.

**Figure 1:**
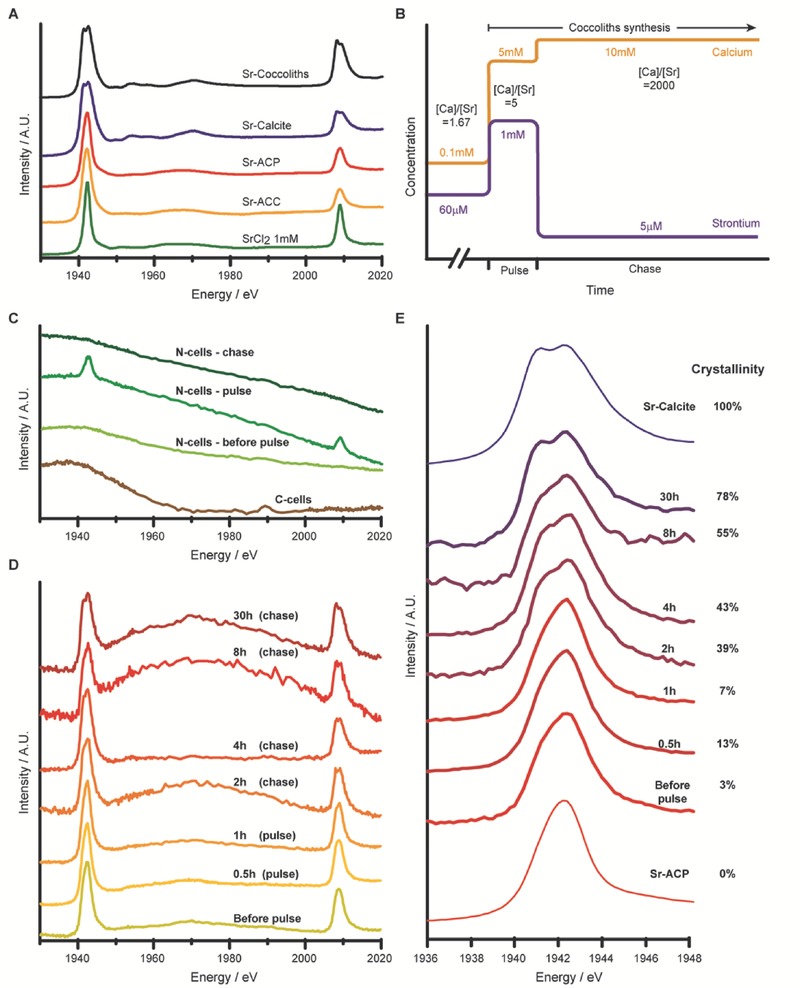
A) Sr L_2,3_‐edge XANES spectra of frozen SrCl_2_ solution, Sr‐doped synthetic ACC, ACP, and calcite, as well as coccolith‐calcite formed in Sr‐enriched medium. B) Schematic representation of Sr and Ca concentrations during the pulse‐chase experiments. C) Spectrum of calcifying cells (C‐cells) grown in standard medium, and decalcified with EDTA prior to freezing, and spectra of Sr pulse‐chase experiment performed on N‐cells, which do not calcify. D) Sr L_2,3_‐edge XANES spectra of pulse‐chase experiments on calcifying cells raised in medium ceasing calcification (low‐Ca medium). Calcification was induced through increasing the Ca concentration together with the Sr pulse. The samples were measured before the pulse and at the indicated time points after the pulse. The undulating background, which is of the same magnitude as the Sr signal (see Figure S3 in the Supporting Information), is due to the uneven sample geometry. E) Detailed scan of the L_3_‐edge of the same samples as in (D), together with spectra from Sr‐doped reference samples. The crystallinity of each sample was determined by linear combination fitting of the spectrum to the two reference spectra of Sr‐ACP and Sr‐calcite (see also Figure S4 in the Supporting Information).

In order to measure XANES spectra of intracellular Sr pools during the process of calcification, cell samples were snap‐frozen. Briefly, the cells were collected on a membrane filter. The filter, containing cells as well as small amounts of medium trapped between them, was immediately frozen in liquid nitrogen and kept at cryo‐conditions during sample mounting and the measurement. This sample preparation ensures that Sr remains in its native environment for the XANES measurement, and that the contribution of extracellular Sr to the signal is minimal. In order to follow a defined quantum of Sr along the mineralization process, we used a pulse‐chase strategy comprising of three Sr concentrations (Figure 1B): before the pulse, the cells were grown in [Sr] = 60 µm, a concentration reported for seawater.^25^ For the pulse, Sr was added to the growth medium to yield [Sr] = 1 × 10^−3^ m. At the end of the 1 h pulse, the cells were pelleted and most of the supernatant was replaced with Sr‐free medium, yielding [Sr] ≈ 5 µm for the chase period.

We tested our experimental approach with two controls. The first was a pulse‐chase experiment of *E. huxleyi* strain CCMP2090, which is unable to produce coccoliths so the cell surface remains naked (N‐cells). The spectra of N‐cell samples before the pulse and 1 h into the chase period showed no Sr peaks, demonstrating the absence of detectable Sr pools (Figure 1C). A sample collected during the pulse showed Sr spectrum similar to that of soluble ions. From this experiment, involving cells that do not calcify, we concluded that our measurements can detect Sr in the medium trapped between cells, but only at the relatively high concentration of the pulse period. We collected data for the nearby phosphorous K‐edge together with the Sr L‐edge (Figure S1, Supporting Information). As phosphorous is a ubiquitous element in all organisms, the simultaneous acquisition of phosphorous spectra served as an internal control confirming that absence of Sr signal is because the cells are Sr‐free and not because of lack of cells in the X‐ray beam.

A second control was to measure calcifying cells (C‐cells) grown in standard medium ([Sr] = 60 µm [Ca] = 10 × 10^−3^ m). Before sampling, the extracellular coccoliths of these cells were completely dissolved to avoid signal from Sr in the extracellular coccolith shell. The absence of an Sr signal from this sample demonstrates that the amount of intracellular Sr taken up by the cells under [Sr] = 60 µm and [Ca] = 10 × 10^−3^ m is insufficient to produce a detectable signal (Figure 1C). Taken together, these controls clearly demonstrate that our experimental approach will not detect Sr of the medium at the concentrations used before the pulse and during the chase, as well as intracellular Sr taken up by cells grown in standard medium.

For the main objective of the study, we performed pulse‐chase experiments on cells that grew for many generations in a medium containing only 1% of the Ca concentration of standard medium ([Ca] = 100 µm. At this concentration cells divide but calcification is inhibited, thus the cells remain free of calcite.^13^ The pulse‐chase experiment started with the induction of coccolith synthesis by elevating the Ca concentration (see Ca trace in Figure 1B), and the simultaneous addition of Sr to a final concentration of 1 × 10^−3^ m for the Sr pulse. With the induction of the calcification process in the calcium‐deplete cells the uptake of Ca and thus also Sr is likely operating at maximum capacity. The chase period started 1 h after Sr addition by replacing the Sr‐rich medium with Sr‐free medium. First intracellular coccoliths could be seen by polarized light microscopy 2 h after the beginning of the experiment (1 h into the chase period). After 4 h, about half of the cells had one or more coccoliths on their surface, and after 30 h, ≈80% of the cells recovered complete coverage with coccoliths (Figure S2, Supporting Information).

The Sr L‐edge XANES spectra collected along the pulse‐chase experiments were somewhat noisy due to the extremely low amounts of Sr. Nevertheless, a gradual transition from single to split peaks is clearly evident (Figure 1D; Figure S3, Supporting Information). The L_3_ energy range was scanned with longer acquisition times (Figure 1E), yielding much better signal‐to‐noise ratio. The crystallinity of Sr in the cells was quantified by performing linear combination fitting using reference spectra of synthetic Sr‐doped ACP and calcite (Figure 1E; Figure S4, Supporting Information). The fits show that the fraction of Sr ions incorporated to a crystalline phase is increasing with time. The initial Sr environment in the cells is similar to Sr in an amorphous phase, but as calcification commences, the signal becomes similar to Sr incorporated into calcite. The intensity of the Sr signal did not markedly change along the chase period (Figure 1D; Figure S3, Supporting Information). This is in agreement with no detectable amounts of Sr being added to the forming coccoliths during the chase period. Thus, the changes in the Sr spectra during the chase period provide a direct demonstration that an intracellular Sr pool is transformed from an amorphous environment to a calcitic environment.

We then imaged *E. huxleyi* cells to correlate our spectroscopic results with the intracellular localization of Sr. Thin slices of high‐pressure‐frozen and freeze‐substituted, resin‐embedded cells were prepared by focus‐ion beam (FIB) milling.^26^ Scanning transmission electron microscopy energy dispersive X‐ray (STEM‐EDX) analyses of cells grown in Sr‐enriched medium could detect intracellular Sr. Small Sr peaks were clearly detected in the coccolith calcite, and also in the Ca–P‐rich bodies where the Sr signal was weak but clearly above the noise level (**Figure** **2**A–C). Sr was not detected in other intracellular locations.

**Figure 2:**
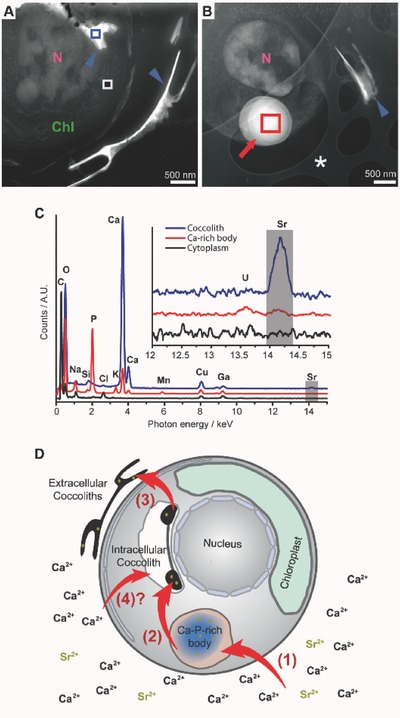
High‐angle annular dark‐field (HAADF) STEM images of FIB‐sectioned cells showing: A) extracellular and intracellular coccoliths (blue arrowheads) and B) Ca–P‐rich body (red arrow). In addition, the nucleus (N), the chloroplast (Chl), and the holey carbon support film (*) are indicated. C) STEM‐EDX spectra of the cytoplasm, Ca–P‐rich body, and coccolith calcite normalized to the Ga peak, taken from the areas in (A) and (B) indicated by rectangles of the corresponding color code. The inset shows a detailed view of the Sr K‐edge energy range. D) A model of the intracellular Ca‐pathway in *E. huxleyi*. Soluble Ca and Sr ions are taken by the cell from the environment and concentrated at the Ca–P‐rich body (1). This compartment serves as an intracellular pool that transports ions to the coccolith vesicle (2), where the calcium carbonate crystals form. Once completed the coccolith is extruded to the cell surface (3). Other pathways transferring Ca and Sr from the environment into the coccolith vesicle (4) may participate in coccolith formation.

Taken together, our results show that Sr XANES at cryo‐conditions, combined with analytical TEM, can monitor Sr transport through intracellular pools in *E. huxleyi* (Figure 2D). The Ca–P‐rich body of *E. huxleyi*, a membrane‐bound pool of amorphous Ca, can accommodate Sr^2+^, which is chemically similar to Ca^2+^, and can pass membranes through Ca channels and thus most likely enters the Ca–P‐rich body via the same transporters as does the Ca. The lack of detectable Sr inside cells grown for several generations in standard medium (Figure 1C—“C‐cells”) and the low Sr content of the bodies in cells grown in Sr‐enriched medium demonstrate that the body is not a permanent sink for Sr, as otherwise Sr would accumulate in the body over cultivation time. An important question, which currently we can only speculate about, is what the amorphous Sr phase in the body is. One possibility is that Sr ions are complexed by polyphosphates, which are suggested components of the body.^13^ In line with this scenario is the colocalization of large amounts of P (Figure 2C). However, in the blue‐green alga *Plectonema boryanum* Sr accumulated preferentially in P‐free bodies demonstrating that another scenario is also possible.^27^ The transformation of the intracellular Sr pool from an amorphous environment to a calcitic environment upon induction of calcite formation demonstrates the transfer of Sr into the coccolith vesicle. Underlying this interpretation are the observations that Sr is clearly incorporated into coccolith calcite (Figure 2C) and that there are currently no indications for the crystallization of calcite inside the body compartment.^13^ Given that no Sr‐specific transporters or binding proteins are known but Ca transporters have been demonstrated to be leaky for Sr, the transfer of Sr from the body into the coccolith vesicle must co‐occur with the transfer of Ca. Therefore, our results also demonstrate the delivery of calcium ions from one dense phase that is phosphorous‐rich to another which is carbonate‐rich. At what stage the carbonate ions are introduced and calcite is forming still remains unclear. The here presented data, but also previous studies, support a crystallization pathway where calcium and carbonate are delivered separately into the coccolith vesicle.^20^, ^21^

One surprising observation from this study is that cells growing in low‐Ca medium concentrate a considerable amount of intracellular Sr (Figure 1D—“before pulse”). This can be the result of the relative abundance of Sr in the low‐Ca medium when the cells unsuccessfully try to accumulate Ca.^28^ This phenomenon was useful for our experimental purpose as it adds to the amount of Sr that is followed during the chase period.

Using Sr‐XANES in pulse‐chase experiments as a proxy for calcium, successfully demonstrated that the Ca–P‐rich body of *E. huxleyi* serves as an ion pool for coccolith formation. That trace metals become incorporated into coccolith calcite is well known but the pathways by which these ions are transported from the seawater into the coccolith vesicle are still unknown.^29^, ^30^, ^31^ The pulse‐chase approach presented here, combined with cryo‐spectroscopy and electron imaging, may be adaptable for clarifying the intracellular pathways of other trace metal impurities in coccolith calcite. We believe that the combination of these techniques will help elucidating the pathways underlying coccolith biomineralization, which is of great importance due to their relevance to various chemical, environmental, and geochemical questions.

## Conflict of Interest

The authors declare no conflict of interest.

## Supporting information

Supplementary
