# Supplementary material for: Trace‐Element Incorporation into Intracellular Pools Uncovers Calcium‐Pathways in a Coccolithophore
Source: Adv Sci (Weinh). 2017 Jul 5;4(10):1700088. doi: 10.1002/advs.201700088 (PMC5644232; doi:10.1002/advs.201700088)
Supplement: Supplementary file 1 — Supplementary [file ADVS-4-na-s001.pdf]

Copyright WILEY-VCH Verlag GmbH & Co. KGaA, 69469 Weinheim, Germany, 2017.

ADVANCED SCIENCE
Open Access

Supporting Information

for *Adv. Sci.,* DOI: 10.1002/advs.201700088

Trace-Element Incorporation into Intracellular Pools Uncovers Calcium-Pathways in a Coccolithophore

*Assaf Gal, Sanja Sviben, Richard Wirth, Anja Schreiber, Benedikt Lassalle-Kaiser, Damien Faivre, and André Scheffel**

Copyright WILEY-VCH Verlag GmbH & Co. KGaA, 69469 Weinheim, Germany, 2016.

Supporting Information

Trace-element incorporation into intracellular pools uncovers calcium-pathways in a coccolithophore

*Assaf Gal, Sanja Sviben, Richard Wirth, Anja Schreiber, Benedikt Lassalle-Kaiser, Damien Faivre, André Scheffel**

Experimental section

Synthetic reference materials

Sr-doped calcite was prepared by mixing 1 ml solution containing 90 mM $CaCl_2$ and 10 mM $SrCl_2$ with 1ml of 100 mM Na_2CO_3 (reagents by Sigma-Aldrich). The mixture was let to ripe for 3 days during which the initial amorphous precipitate crystalized. Sr-doped amorphous calcium carbonate was prepared by mixing 1 ml solution containing 90 mM $CaCl_2$ and 10 mM $SrCl_2$ with 1 ml solution containing 100 mM Na_2CO_3 and 0.9 M NaOH. The mixture was immediately washed with ethanol and vacuum dried to prevent crystallization. Sr-doped amorphous calcium phosphate was prepared by mixing 1 ml solution containing 90 mM $CaCl_2$ and 10 mM $SrCl_2$ with 1 ml solution containing 100 mM Na_2HPO_4. The mixture was immediately washed with ethanol and vacuum dried to prevent crystallization. The phase (amorphous or crystalline) of the precipitates was determined using polarized light microscopy and ATR-FTIR spectrometry (Figure S5).

Cells, strains, and culturing

Emiliania huxleyi strains AWI1516 (Alfred Wegner Institute, Germany), which produces coccoliths, and CCMP2090 (National Center for Marine Algae and Microbiota, USA), which

does not produce coccoliths, were grown in artificial seawater medium Aquil at 18 °C and a 12/12 h light/dark cycle, as described before.[13] The concentration of nitrate in the medium was 0.2 mM and of phosphate 10 µM. The standard medium contained 10 mM $CaCl_2$ and 60 µM $SrCl_2$. Cells devoid of extracellular coccoliths were obtained by adding 1 / 50 volume of 0.5 M EDTA pH = 8.0 to the culture. This treatment dissolved extracellular calcite, but the calcite of intracellular coccoliths remained undissolved. Cultures of cells growing at low-Ca medium (0.1 mM $CaCl_2$) were grown for more than 6 months, these cultures continued to divide whereas coccolith formation was ceased.

Sample preparation for cryoXANES

For pulse-chase experiments 50 ml of cell culture were placed at 14 °C during the dark period prior to the experiment. The entire pulse-chase experiment was conducted at 14 °C since the low temperature slowed down the calcification process while keeping the same percentage of fully calcifying cells after 24 h. At the beginning of the pulse the cells were exposed to light and the appropriate volumes of 1 M $CaCl_2$ and 100 mM $SrCl_2$ were added to the culture. With the exposure to light the cells resumed coccolith formation, which at the end of the dark phase is ceased. After 50 minutes, the cells were pelleted at 5000 g, 14 °C, for ten minutes. 49.8 ml of the medium were decanted, and 49.8 ml Sr-free medium was added to the remaining 0.2 ml of culture containing the cells. The cells were re-suspended and left at continuous light conditions. In order to freeze a sample 10 ml cell culture were removed and pelleted at 5000 g, 4 °C, for ten minutes. 9.5 ml of the supernatant were decanted. The 0.5 ml medium containing the cells was filtered using an Isopore™ membrane filters, 100 nm pore size (Merck Millipore, Germany). The membrane filter with the cells was blotted dry and immediately frozen in liquid nitrogen.

cryoXANES measurements

The frozen filters were attached to a frozen copper sample holder using two metals rings screwed to the holder. The XANES spectra of the Sr L-edge and P K-edge were acquired at the LUCIA beamline at the SOLEIL Synchrotron Light Source (Gif-sur-Yvette, France) by scanning the X-ray beam energy from 1900-2200 eV in 1.0 eV steps before the Sr L_3-edge and between the Sr L_3-edge and L_2-edge. The Sr absorption edges were scanned with 0.2 eV steps, and the entire P K-edge was scanned with 2 eV steps. All samples were measured under vacuum and at cryo-conditions. The synchrotron ring energy was 2.75 GeV and the current was up to 400 mA. The X-rays energy was selected by a double crystal Si(111) monochromator, which was calibrated using Ti foil and setting the edge at 4966 eV. The beam size on the sample was approximately 0.5 × 2 mm^2. In order to improve the signal-to-noise ratios, 3 scans of the full Sr $L_{3,2}$-edge spectra were averaged. 10 scans were averaged for the Sr L_3-edge spectra.

Data analysis and fitting

For each Baseline subtraction, normalization, and data processing and analysis were performed using the Athena software package4.0. Normalization was performed using a linear pre-edge function between 25 eV and 5 eV below E_o (set to 1940 eV) and a linear post-edge function between 10 eV and 60 eV above E_0. Linear combination fitting was performed on spectra using two standards, Sr-doped ACP and calcite.

STEM imaging and EDX

Cells growing for 24 h at high-Sr medium ([Sr] = 600 µm, [Ca] / [Sr] = 16.7) were high-pressure frozen with 1-hexadecane (Sigma-Aldrich) as cryoprotectant and freeze-substituted with 0.25 % (v/v) glutaraldehyde (Agar Scientific), 0.01 % (w/v) uranyl acetate (Sigma-Aldrich) and 8 % (v/v) 2,2-dimethoxypropane (Sigma-Aldrich) in acetone for 5 days at -85 °C. Samples were warmed-up to room temperature over 2 days and embedded in Spurr's

resin over 4 days. Thin sections were prepared using the focused ion beam (FIB) technique. The FIB device was a FEI FIB 200 single beam unit with a Ga ion beam and an acceleration voltage of 30 kV. The sputtered FIB foils had an approximate size of 15 x 8 x 0.250 µm and were placed on a copper grid with a holey carbon film. High-angle annular dark-field electron imaging (HAADF) and EDX spectroscopy were performed on a Tecnai G2 F20 X-Twin microscope equipped with a field-emission electron source (operated at 200 kV), a Fishione HAADF detector at 330 mm camera length for image acquisition in the STEM mode and an EDAX Genesis X-ray analyzer.

Supplementary Figures

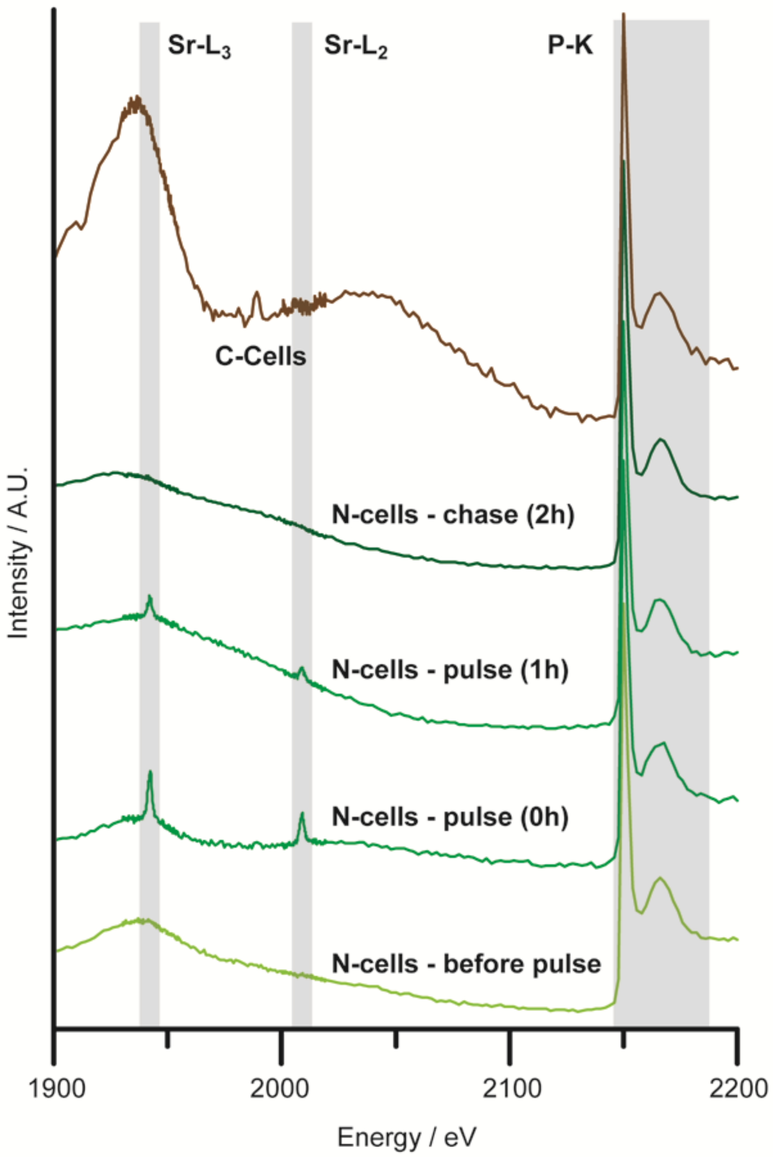

Figure S1. Sr $L_{3,2}$-edge and P K-edge XANES spectra of the same samples as in Figure 1c. The spectra were normalized to the phosphorus edge white-line as an approximation for cellular material in the sample. The two pulse samples, at the beginning of the pulse (0 h) and just before washing (1 h), show that Sr is not incorporated into the cells during this period.

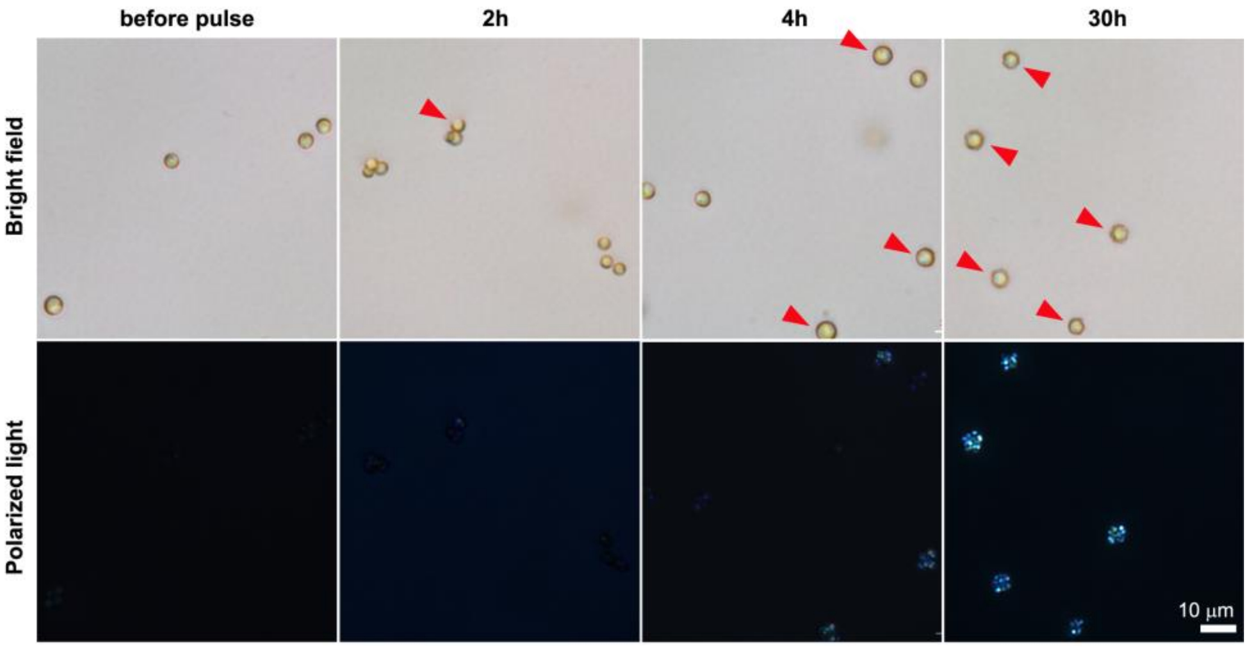

Figure S2. Light microscopy images of *E. huxleyi* cells during a pulse-chase experiment. The cells were growing in low-Ca medium before the pulse. The indicated time is after the start of the one hour pulse. Each sample was imaged in bright field and with crossed polarizers to detect the birefringence of the calcite crystals. Red arrows indicate cells with detectable birefringence.

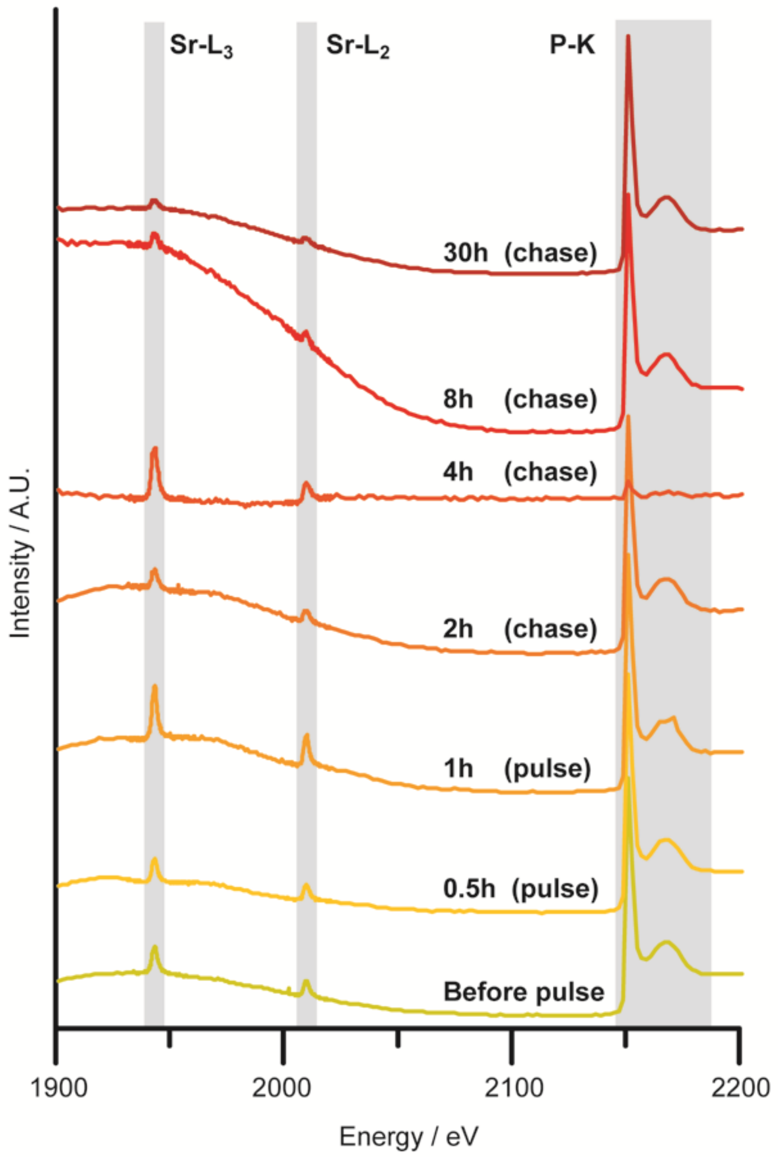

Figure S3. Sr $L_{3,2}$-edge and P K-edge XANES spectra of the same samples as in Figure 1d. The spectra (except for the 4 h) were normalized to the phosphorus edge 'white-line' as an approximation for cellular material in the sample. The undulating background, which is of the same magnitude as the Sr signal, is due to the uneven sample geometry.

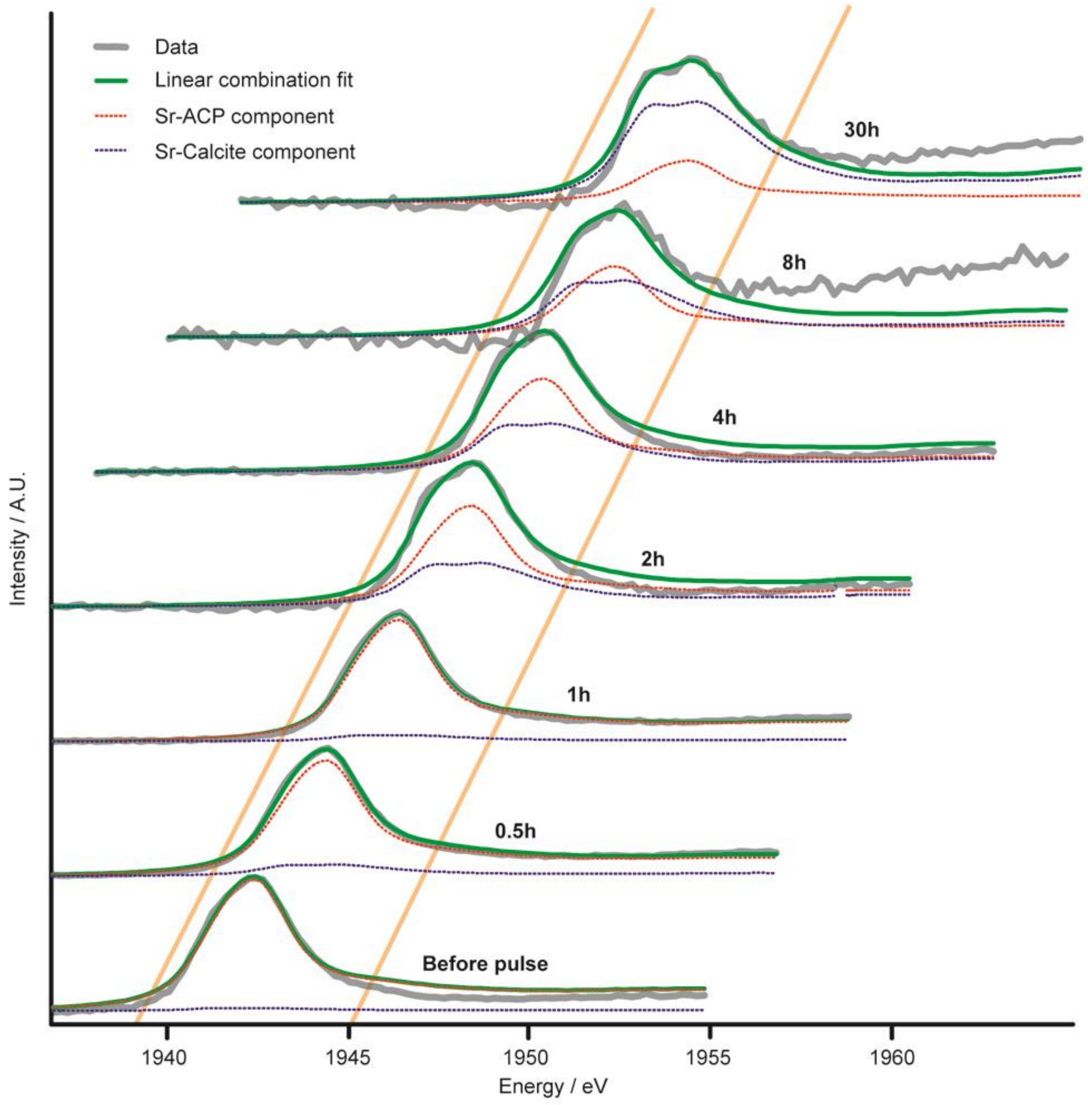

Figure S4. The different components of the linear combination fitting analysis for the spectra shown in Figure 1e. The raw data of the Sr L_3-edge spectra are shown, as well as the resulting fits and the relative components of the Sr-ACP fraction and the Sr-calcite fraction. Two orange lines indicate the data range that was considered for achieving the best fit. See also Table S1.

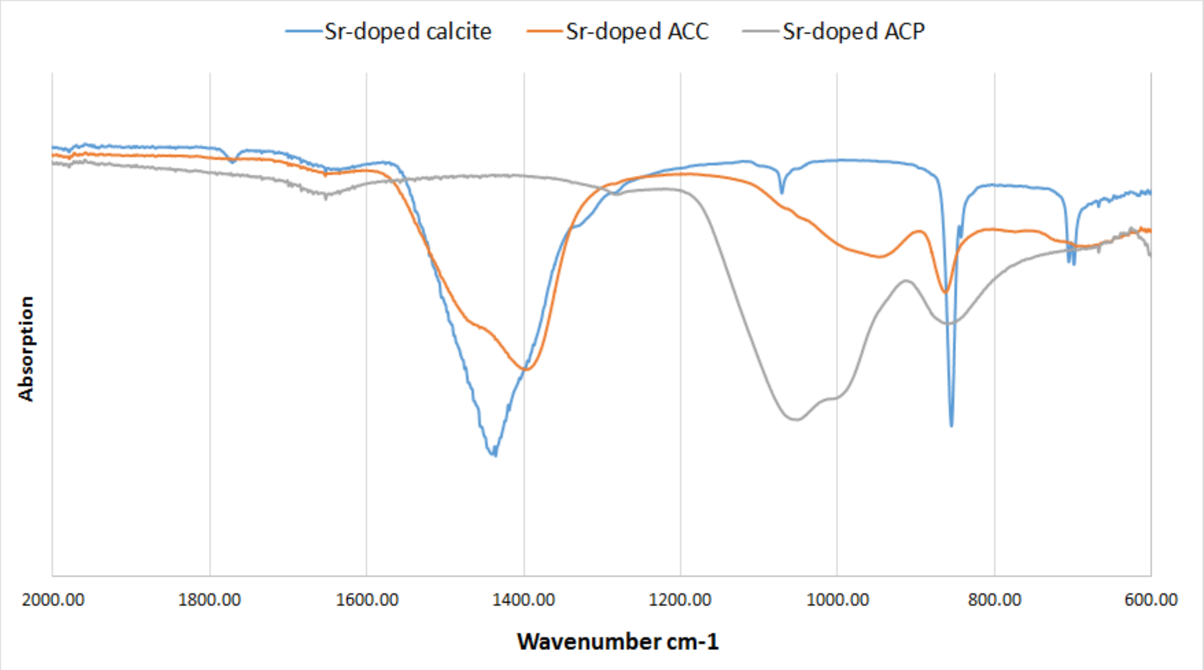

Figure S5. FTIR spectra of Sr-doped calcite, ACC, and ACP. The amorphous nature of the Sr-doped ACC and ACP samples is evidenced by the characteristic position of the carbonate ν2 peak at 863 cm^{-1} and ν3 of phosphate at 1045 and 990 cm^{-1}.

Table S1: Fitting parameters for the linear combination fit presented in Fig S4

		Sr-ACP				Sr-calcite				
Sample	scaleby	weight	error	e0	error	weight	error	e0	error	crystallinity
Before pulse	1.378	1.3355	0.0615	0.0625	0.0287	0.0422	0.0728	0.0922	1.4823	3%
0.5h	0.996	0.8707	0.0185	0.0345	0.0133	0.1252	0.0218	0.1099	0.1507	13%
1h	0.981	0.9128	0.0215	0.0439	0.0147	0.0678	0.0255	0.0950	0.3240	7%
2h	1.31	0.8016	0.0630	0.0524	0.0487	0.5088	0.0746	0.0475	0.1261	39%
4h	1.476	0.8405	0.0679	0.0377	0.0502	0.6353	0.0804	0.0429	0.1090	43%
8h	0.334	0.1514	0.0286	0.2293	0.1194	0.1828	0.0339	0.4589	0.1639	55%
30h	0.458	0.0989	0.0228	0.0653	0.1453	0.3589	0.0270	0.1663	0.0653	78%

For each sample the scaling factor (‘scaleby’) used for the fitting is showed. For each of the two reference spectra the weight given in the fit and calculated error is shown, as well as the difference applied to the E0 value and its error.
